# Aortic dissection presenting with Hemoptysis: a diagnostic challenge

**DOI:** 10.1093/omcr/omaf107

**Published:** 2025-07-27

**Authors:** Ahmad Alashqar, Mohammad Alashqar, Ahmad Daraghmeh, Hala A I Dalbah, Mohammed I Abu Kamesh, Yazan Giacaman, Salma Hajj-Qasem

**Affiliations:** Department of Medicine, Alexandria University, Alexandria, Egypt; Department of Medicine, An-Najah National University, PO Box 7, Nablus, West Bank, Palestine; Department of Medicine, An-Najah National University, PO Box 7, Nablus, West Bank, Palestine; Department of Internal Medicine, Tubas Turkish Governmental Hospital, Tubas, Palestine; Department of Internal Medicine, Tubas Turkish Governmental Hospital, Tubas, Palestine; An-Najah National University Hospital, Asira Street, Nablus, West Bank, Palestine; Department of Medicine, An-Najah National University, PO Box 7, Nablus, West Bank, Palestine; Department of Medicine, An-Najah National University, PO Box 7, Nablus, West Bank, Palestine

**Keywords:** Hemoptysis, aortic dissection, Stanford type B

## Abstract

Hemoptysis is the expectoration of blood originating from the lower respiratory tract and has a wide differential diagnosis, ranging from benign conditions to life-threatening diseases that include pulmonary embolism, severe infections, and aortic dissection. We present a 53-year-old male with a history of hypertension, COPD, and smoking, presenting to the emergency department with a two-day history of hemoptysis. In spite of the absence of classical symptoms such as chest pain, his chest X-ray showed mediastinal widening, and further imaging was performed with contrast-enhanced CT angiography. The CT scan confirmed a Stanford Type B aortic dissection. After multidisciplinary review, medical management was decided upon with antihypertensive medications and tranexamic acid. The patient remained hemodynamically stable and was transferred for further management. This case indicates the inclusion of aortic dissection in the differential diagnosis of hemoptysis, even without classic symptoms.

## Introduction

Hemoptysis can be defined as the expectoration of blood coming from the lower respiratory tract [1]. It presents with a long differential diagnosis. Common causes include bronchiectasis, bronchitis, pulmonary tuberculosis, and neoplasms of the lung [2]. Generally, it is often not-life-threatening. However, it often could be part of the presenting signs of life-threatening condition, such as pulmonary emboli, severe pulmonary infection, diffuse alveolar bleeding, or dissecting of the aorta [3].

Herein, we describe a case of a 53-year-old male diagnosed with an aortic dissection presenting to the emergency department with a history of hemoptysis lasting for 2 days without chest pain. The current case describes a less common presentation of an aortic dissection and the importance of its inclusion in the differential diagnosis of hemoptysis of undetermined origin.

## Case description

A 53-year-old male, a heavy smoker, presented to the emergency department with a 2-day history of hemoptysis, describing the expectoration of large amounts of fresh blood approximately 250 ml (equivalent to a cup of water); some was visible on tissues brought in by the patient. He described similar episodes two and four years ago, both of which resolved spontaneously without medical intervention. His past medical history was significant for hypertension, COPD, and epiglottic hypertrophy surgery 4 years ago. He denied the use of any anticoagulant or antiplatelet agent and also denied a history of fever, chest pain, abdominal pain, vomiting, hematemesis, weight loss, night sweats, and anorexia. There were no significant family medical history, and he denied the use of alcohol.

On arrival, he was hemodynamically stable with a blood pressure of 140/75 mmHg in the right arm and 130/69 mmHg in the left arm, pulse rate of 63 beats per minute, and oxygen saturation of 91% on room air. He was afebrile. Physical examination revealed clear lung fields with good bilateral air entry, no murmurs on cardiac auscultation, and a soft nontender abdomen.

The lab investigation included tests for INR, aPTT, D-dimer, C3 and C4 complement levels, creatinine, sodium, potassium, random blood glucose and CRP. Also, a CBC was done to assess WBC, hemoglobin, RBC, hematocrit, MCV and platelets. (Table 1).

**Table 1:** Lab investigation.

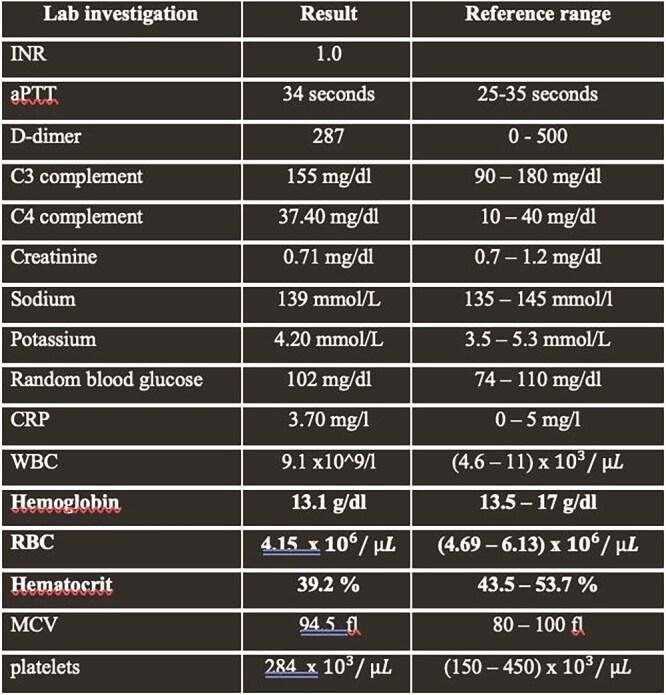

**Figure 1:**
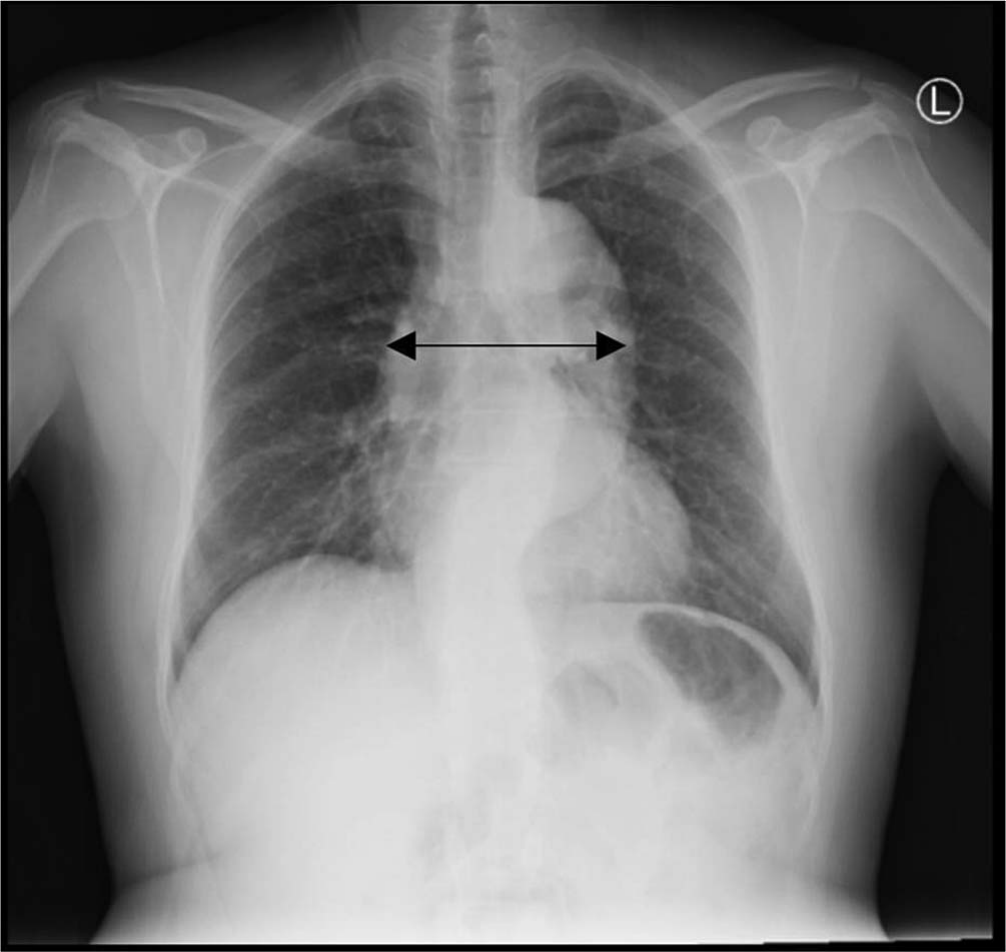
Chest X-ray revealed a widened mediastinum.

**Figure 2:**
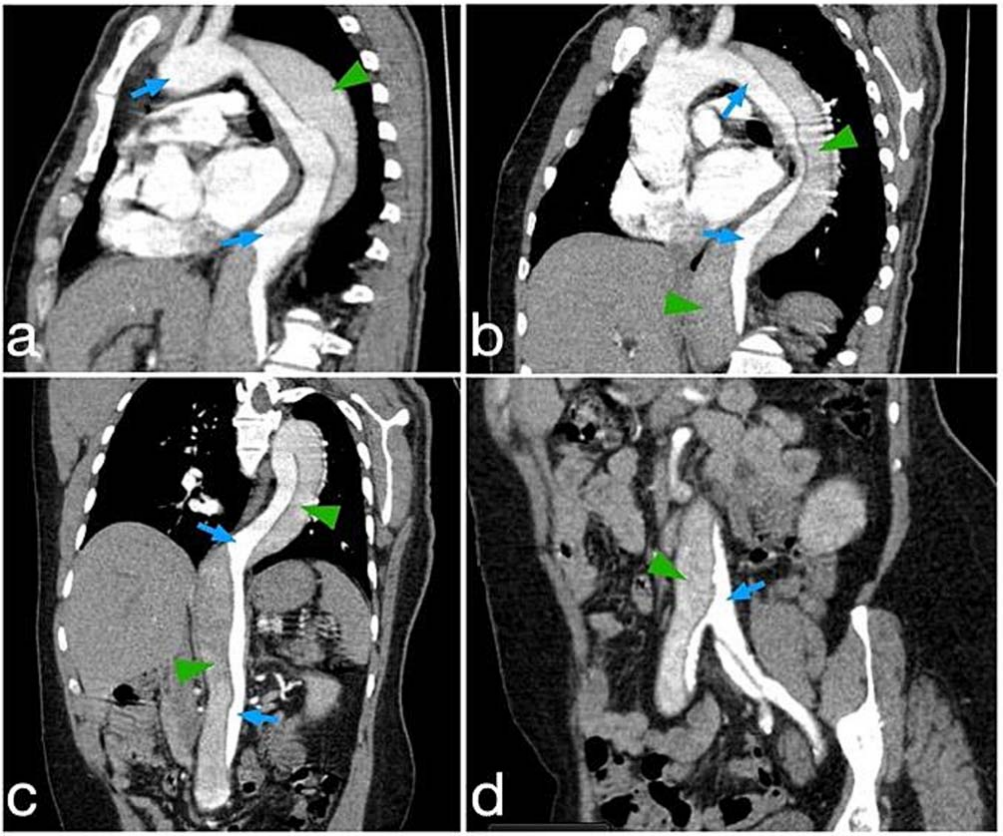
Reformatted CT angiography images demonstrate different levels of the aortic dissection: (a and b) at the level of the aortic arch and thoracic aorta, (c) at the level of the thoracoabdominal aorta, and (d) at the level of the aortic bifurcation.

A chest X-ray showed mediastinal widening **(**Fig. 1**)** and thus further evaluation with contrast-enhanced CT aortic angiography was performed. The CT scan identified a Stanford type B aortic dissection with an intimal flap extending from just distal to the left subclavian artery to the abdominal aorta and into both common iliac arteries, predominantly on the right side. The abdominal aorta demonstrated aneurysmal dilatation, which measuring 4.2 cm in anterior–posterior diameter, with a smaller true lumen and a larger false lumen. The celiac trunk, superior mesenteric artery, and left renal artery arise from the true lumen (Fig. 2).

Consultation with cardiothoracic surgery and interventional radiology services was obtained. After a thorough review, it was determined that this patient would be a poor candidate for surgical or endovascular treatment due to a high perioperative risk related to anatomical complexity combined with comorbid conditions. Thus, a multidisciplinary treatment plan was implemented, including medical management with antihypertensive medications amlodipine 5 mg by 2, bisoprolol 5 mg once, losartan 50 mg once. In addition to, tranexamic acid 500 mg IV every 8 h, and tranexamic acid nebulizer 500 mg three times daily.

The patient remained hemodynamically stable during the course of his hospital stay, we observed blood pressure, the target blood pressure is a systolic pressure of 100–120 mmHg and ensured that the heart rate did not exceed 60 beats per minute. Additionally, we observed for hemoptysis. The pulmonologist refused to perform a bronchoscopy.

## Discussion

The initial assessment of hemoptysis, history, and physical examination should be directed at the diagnosis of its most common causes. Once these have been excluded, the investigation should proceed to less common causes. Among the rare causes, one should know clinically life-threatening disorders such as complicated lung infection, for example, lung abscess, necrotizing pneumonia, and immunological disorders involving pulmonary vasculature that include Goodpasture syndrome and granulomatosis with polyangiitis. Other conditions included are pulmonary embolism, aortobronchial fistulas, and others based on clinical suspicion.

Aortic dissection, however uncommon, is a catastrophic vascular condition caused by a tear in the aorta’s intimal layer, resulting in the separation of the aortic wall layers. Blood enters between the intima and media, spreading the dissection either proximally or retrogradely, resulting in reduced blood supply to vital organs [4]. An aortic rupture is a complete tear through all three layers of the aorta. While the classic presentation of acute aortic dissection is sudden, intense, ‘tearing’ chest pain, milder symptoms sometimes result in missed diagnoses. Despite the literature, many aortic dissections go undetected in the emergency department; approximately 15% to 43% of verified cases are correctly diagnosed on the first visit [5–7]. Without medical or surgical, fatality rates surpass 50% within 48 h of symptom onset. Despite its rarity, acute aortic dissection requires immediate detection and multidisciplinary healthcare management, with improved outcomes found in high-volume centres using experienced teams, ‘aorta code’ protocols, and specialised aortic centres [4].

**Figure 3:**
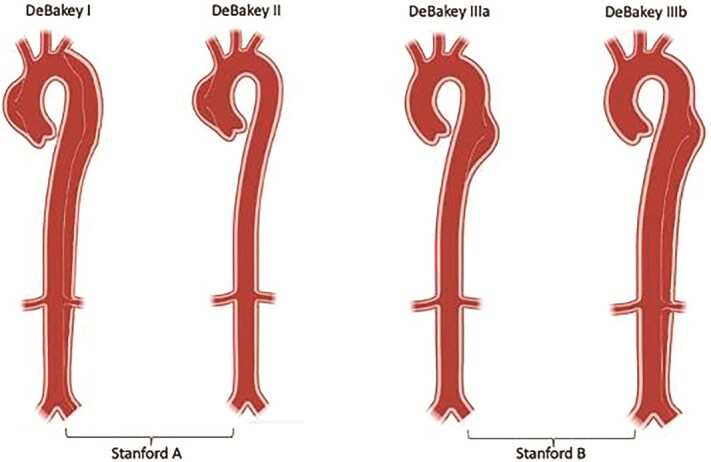
DeBakey and Stanford aortic dissection classification.

The Stanford classification divides aortic dissections into two types according to whether the ascending or descending aorta is involved. Stanford Type A: this involves the ascending aorta independent of the primary intimal tear. It is classically defined as a dissection occurring proximal to the brachiocephalic artery. However, Stanford Type B: This originates distal to the left subclavian artery and only involves the descending aorta (Fig. 3). On the other hand, DeBakey further classifies dissections into three types based on origin and extent: DeBakey Type I: Originates in the ascending aorta and extends into the aortic arch and into the descending aorta, DeBakey Type II: Limited to the ascending aorta, or DeBakey Type III: Originates in the descending aorta and extends distally, either above the diaphragm (Type IIIa) or below the diaphragm (Type IIIb, as seen in our patient.) [8].

Multiple high-risk predisposing factors are related to nontraumatic aortic dissection. The most common of these is hypertension, which may be found in approximately 70% of patients with distal Stanford Type B dissections. Abrupt and severe elevations in blood pressure may also precipitate dissection in the setting of such activities as strenuous weightlifting or with the use of sympathomimetic agents including cocaine, ecstasy, or energy drinks [4]. Genetic disorders greatly increase the risk and include Marfan syndrome, Ehlers-Danlos syndrome, Turner syndrome, and bicuspid aortic valve. For instance, Marfan syndrome is seen in 50% of patients with aortic dissection who are under 40, compared to only 2% in older individuals, and is associated with cystic medial necrosis of the aortic wall [4]. Other risk factors include coarctation of the aorta, preexisting aortic aneurysm, and atherosclerosis. Pregnancy and delivery, particularly in disorders of connective tissue such as Marfan syndrome, further raise the risk. Other contributing factors include family history of aortic dissection, prior instrumentation or surgery of the aorta including coronary artery bypass grafting, valve replacement, or catheter placement, and inflammatory or infectious causes of vasculitis, for example, syphilis [9].

The exact mechanism of hemoptysis in aortic dissection is not known. It has been postulated that the dissection leads to erosion into adjacent structures, including the bronchial tree or pulmonary vessels, leading to bleeding [10]. However, in our patient There is no radiological confirmation of the communication with the bronchial tree and dissection. The absence of other signs and symptoms of aortic dissection made diagnosis difficult and further supports the need to maintain a broad differential diagnosis. Chest X-ray showing mediastinal widening prompted further investigation with CT aortic angiography, which led to confirmation of the diagnosis. Imaging is one of the keystones in the diagnosis of aortic dissection; CT angiography remains the mainstay, due to its high sensitivity and specificity [4].

Management of Stanford type B aortic dissection can be broadly divided into medical, surgical, and endovascular approaches based on the presentation and risk factors of the patients. In this case, medical management was decided upon due to the anatomical complexity and significant comorbidities that presented the patient as a poor candidate for invasive procedures. This also agrees with current guidelines that advocate for medical therapy, including strict blood pressure control, as the primary treatment for uncomplicated type B aortic dissections [7]. The absence of malperfusion or indications of (early) disease development, and the presentation within 14 days of symptom onset, are characteristics of acute uncomplicated Stanford type B aortic dissection [11, 12]. Moreover, the survival rates from 50 to 80% at five years and 30 to 60% at 10 years for medical therapy in non-ruptured aortic dissection [13].

A previous retrospective study following a Ruptured Abdominal Aneurysm reported that, out of 157 patients, 47 received palliative care while 110 underwent surgical intervention. In the surgically treated, the one-year mortality rate was 50% whereas the 30-day mortality rates were 40.8% for endovascular repair and 31.7% for open repair, with no statistically significant difference between the two approaches. It is indicated that 65% of survivors were discharged to their homes, while 34.8% required rehabilitation in nursing facilities. However, all palliative patients died within three days [14]. Our patient presented with a non-ruptured aortic dissection, which usually presents less dramatically than in the ruptured case. The presentations of non-ruptured aortic dissection are quite varied; thus, this hemoptysis was an atypical presentation for the disease. The absence of classical symptoms such as severe chest pain, combined with stable vital signs, made diagnosis difficult. In addition, approximately 10% of aortic dissections are painless, though symptoms may arise as a result of the dissection’s consequences and Painless acute aortic dissection is linked to higher mortality and is more common in patients with type A dissection than type B dissection [11, 12].

## Conclusion

Aortic dissection is an uncommon cause of hemoptysis, and diagnosis may be missed in the absence of other classical symptoms such as chest pain. This case highlights the need for broad differential diagnosis in the workup of hemoptysis, especially among patients with risk factors such as hypertension and smoking. For conditions like these, with respect to patient outcomes, early detection and appropriate management which would be strict blood pressure control are key. Multidisciplinary management is thus the key in the management of aortic dissection and needs a balance between medical and surgical intervention in the light of comorbid conditions and anatomical complexity. Further studies are necessary to better explain the mechanisms of hemoptysis in aortic dissection.

## Data Availability

Not applicable (this manuscript does not report data generation or analysis).
